# Fear and Reward Circuit Alterations in Pediatric CRPS

**DOI:** 10.3389/fnhum.2015.00703

**Published:** 2016-01-19

**Authors:** Laura E. Simons, Nathalie Erpelding, Jessica M. Hernandez, Paul Serrano, Kunyu Zhang, Alyssa A. Lebel, Navil F. Sethna, Charles B. Berde, Sanjay P. Prabhu, Lino Becerra, David Borsook

**Affiliations:** ^1^Department of Anesthesiology, Perioperative, and Pain Medicine, Boston Children’s Hospital, BostonMA, USA; ^2^Department of Psychiatry, Boston Children’s Hospital, BostonMA, USA; ^3^PAIN Research Group, Boston Children’s Hospital, WalthamMA, USA; ^4^Harvard Medical School, BostonMA, USA; ^5^Department of Radiology, Boston Children’s Hospital, BostonMA, USA

**Keywords:** chronic pain, neuropathic pain, children, putamen, functional imaging, anterior cingulate, fMRI, amygdala

## Abstract

In chronic pain, a number of brain regions involved in emotion (e.g., amygdala, hippocampus, nucleus accumbens, insula, anterior cingulate, and prefrontal cortex) show significant functional and morphometric changes. One phenotypic manifestation of these changes is pain-related fear (PRF). PRF is associated with profoundly altered behavioral adaptations to chronic pain. For example, patients with a neuropathic pain condition known as complex regional pain syndrome (CRPS) often avoid use of and may even neglect the affected body area(s), thus maintaining and likely enhancing PRF. These changes form part of an overall maladaptation to chronic pain. To examine fear-related brain circuit alterations in humans, 20 pediatric patients with CRPS and 20 sex- and age-matched healthy controls underwent functional magnetic resonance imaging (fMRI) in response to a well-established fearful faces paradigm. Despite no significant differences on self-reported emotional valence and arousal between the two groups, CRPS patients displayed a diminished response to fearful faces in regions associated with emotional processing compared to healthy controls. Additionally, increased PRF levels were associated with decreased activity in a number of brain regions including the right amygdala, insula, putamen, and caudate. Blunted activation in patients suggests that (a) individuals with chronic pain may have deficits in cognitive-affective brain circuits that may represent an underlying vulnerability or consequence to the chronic pain state; and (b) fear of pain may contribute and/or maintain these brain alterations. Our results shed new light on altered affective circuits in patients with chronic pain and identify PRF as a potentially important treatment target.

## Introduction

Fear of pain is considered to be a significant process in pain exacerbation and persistence (Wiech and Tracey, 2009, 2013; Flor, 2012; Simons et al., 2014a). Normally, fear occurs as a protective response to a present or potential threat. In the healthy state, fear is elicited by the sense of a threat (Canteras et al., 2008; Cezario et al., 2008), which activates cognitive (Gilmartin et al., 2014), affective (Fernando et al., 2013), and motor brain circuits (Kincheski et al., 2012). As such, these processes activate an individual’s defense system, which, in turn, induces anti-nociception through descending pain modulation. In chronic pain, a number of brain circuits are known to be altered (Bushnell et al., 2013), including those known to be involved in fear (Yu et al., 2014). Fear avoidance stems from individual perception as well as ongoing symptomatic feedback from their clinical condition that may contribute to the chronification of pain (Asmundson et al., 1999; Vlaeyen and Linton, 2000).

A fear circuit has been defined that includes a number of brain regions in the subcortical (e.g., amygdala, hippocampus, thalamus), cortical [e.g., prefrontal cortex (PFC), sensory cortex], and brainstem regions (e.g., locus coeruleus; Tovote et al., 2015). Among these brain areas, the amygdala plays a pivotal role in fear processing (Sears et al., 2014). Human imaging studies examining common pathways implicated in both fear and pain processing implicate affective brain circuits that include the PFC, insula, anterior cingulate cortex (ACC), hippocampus, nucleus accumbens, and amygdala (Bushnell et al., 2013; Mansour et al., 2014). In an effort to integrate clinical phenotypes of altered cognitive–affective states with underlying brain mechanisms, recent studies have focused on pain catastrophizing and pain-related fear (PRF) in chronic pain patients. Among the reported findings, enhanced medial PFC-default mode network functional connectivity has been associated with higher levels of pain rumination in chronic pain patients (Kucyi et al., 2014). Additionally, increased pain-evoked activity in the mPFC, cerebellum, ACC, dorsolateral PFC (dlPFC), and claustrum has been associated with heightened pain catastrophizing (Gracely et al., 2004). Another study showed that greater dlPFC volume has been linked to decreased pain catastrophizing after cognitive-behavioral treatment (Seminowicz et al., 2013). In the context of PRF, our group has observed increased resting-state functional connectivity between the amygdala and PFC, middle temporal lobe, ACC, and hippocampus that was associated with higher PRF scores in pediatric CRPS (Simons et al., 2014b). One recent study evaluating the influence of PRF on evoked brain activity to viewing aversive movements reported no differences in fear level between patients and healthy controls (Barke et al., 2012), which may be explained by the method used to elicit fear (Salomons and Davis, 2012).

The aim of the current study was to examine fear perception circuit alterations in pediatric patients with complex regional pain syndrome (CRPS) using a well-established fearful faces paradigm (Shin et al., 2005; Etkin and Wager, 2007) to examine whole-brain evoked responses compared to healthy controls and by PRF level. We hypothesized that patients with CRPS would show altered activation patterns in the amygdala and related fear-circuitry areas (i.e., ACC, insula, PFC, hippocampus) in response to fearful stimuli and that these differences would be enhanced among high-fear patients. To our knowledge, this is the first study to evaluate fear perception circuits in pediatric patients suffering with chronic pain. Given that both hypoactivation (in Panic Disorder; Pielech et al., 2014) and hyperactivation (in Post-Traumatic Stress Disorder; Shin et al., 2005) have been observed, we did not hypothesize directionality of response.

## Materials and Methods

### Participants

Of the 53 patients who were contacted and potentially eligible, 20 patients with CRPS (ages 8–20) were recruited from the Chronic Pain Clinic in the Pain Treatment Service at Boston Children’s Hospital (BCH) for this BCH institutional review board approved study. Written informed consent was obtained from all recruited subjects in accordance with the Declaration of Helsinki. Of the 33 patients not enrolled, 17 declined participation [32% decline rate; reasons included: not interested (Bushnell et al., 2013), overwhelmed/can’t make time commitment (Buhle et al., 2014), did not want an MRI (Becerra et al., 2014), claustrophobic (Asmundson et al., 1999)] and 16 were ineligible [reasons included: mental health or medical comorbidity (Bushnell et al., 2013), permanent metal implants (Buhle et al., 2014), left handed (Barke et al., 2012), no pain (Asmundson et al., 1999)]. Both the patient and parent were consented for the study. Parents were present during the study visit, but not in the scanner room. Patients were included in the study if (1) they refrained from using analgesic medication >4 h prior to the study session, (2) they experienced lower extremity CRPS [based on Budapest criteria; (Harden et al., 2010)] and (3) their average pain intensity was >5 on a 11-point numerical rating scale (NRS). They were excluded if they had (1) claustrophobia, (2) significant medical problems [e.g., uncontrollable asthma and seizures, cardiac diseases, severe psychiatric disorders (e.g., suicidal ideation, PTSD), and neurological disorders other than CRPS], (3) pregnancy, (4) medical implants and/or devices, (5) were taking opioid medication, and (5) weight > 285 pounds which corresponded to the weight limit of the magnetic resonance imaging (MRI) table. Twenty sex- and age-matched healthy controls were recruited in the greater Boston area through advertisements. Each study session consisted of a neurological exam with a study physician, questionnaires, and an MRI scan.

### Measures and Stimuli

#### Emotional Faces Paradigm

**FIGURE 1:**
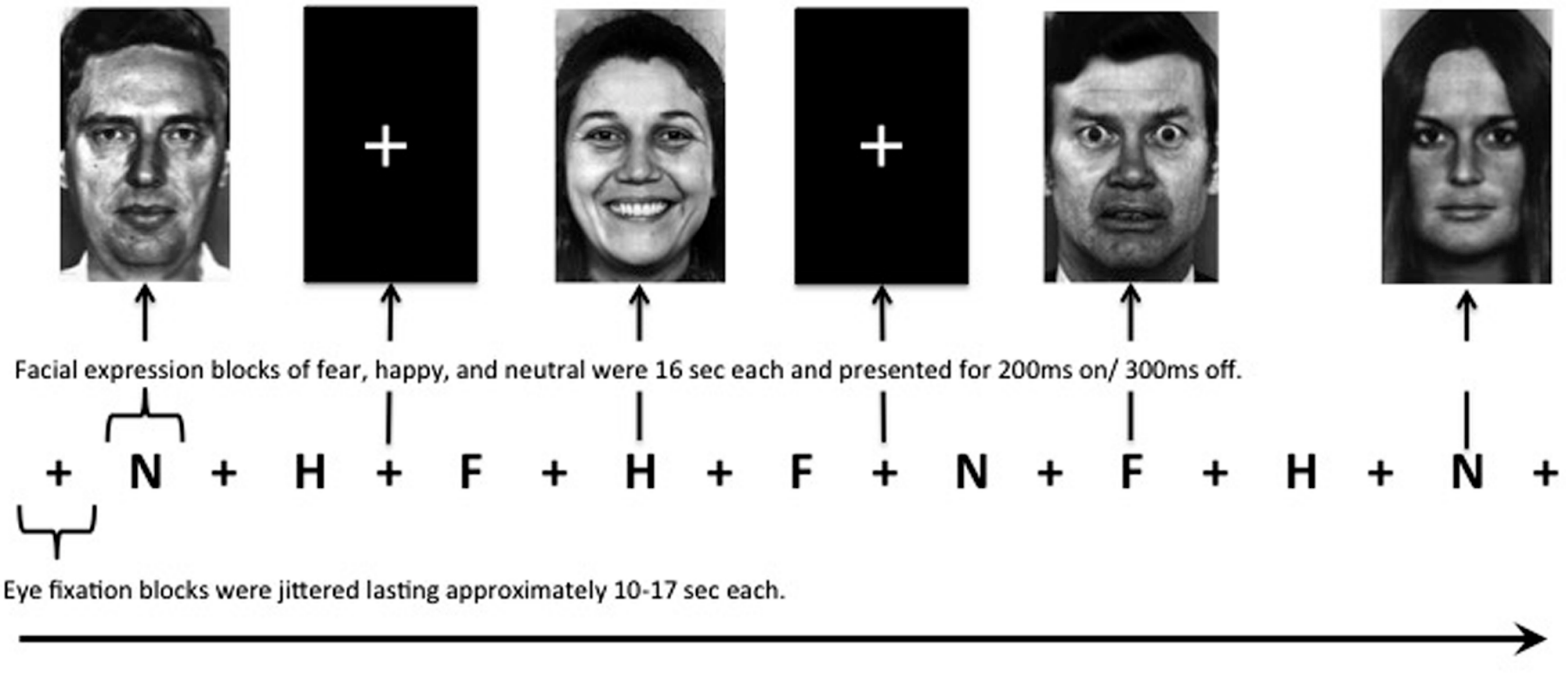
**Facial expression paradigm.** All neutral, happy, and fearful blocks are 16 s in duration with interspersed eye fixation blocks jittered lasting approximately 10–17 s each. Male and female faces in each block were presented in a pseudorandom order such that facial expressions of a single identity are never presented in succession for a total of 32 faces presented in each block. There were two runs. The first run consisted of the following series: +N+H+F+H+F+N+F+H+N+ (as depicted above) and the second run was +N+F+H+F+H+N+H+F+N+. +, eye fixation; N, neutral faces; H, happy faces; F, fearful faces.

Participants viewed standardized gray-scale face stimuli consisting of female and male individuals depicting affective expressions (Ekman and Friesen, 1976), using six happy (H), six fear (F), six neutral (N) stimuli. All faces were matched for overall luminosity and size, and were equally aligned on a black background template. Each face was presented for 200 ms, with a 300-ms interstimulus interval in a pseudorandom order such that facial expressions of a single identity are never presented in succession. Each block of faces was 16 s in duration (e.g., 32 fearful faces were presented in each fear block). Interspersed eye fixation blocks (+) between faces stimuli blocks were jittered lasting approximately 10–17 s each. There were two runs. The first run consisted of the following series of blocks: +N+H+F+H+F+N+F+H+N+ and the second run was +N+F+H+F+H+N+H+F+N+ (see **Figure 1**). This paradigm was modeled after those used in previous studies to evoke fear circuitry (Shin et al., 2005). The facial stimuli were displayed using standardized software (MacStim 3.0; WhiteAnt Occasional Publishing, West Melbourne, Australia). Each run was approximately 5; 10 min for the entire evoked paradigm. Immediately after each scanning session, participants were asked to rate the facial expressions on scales of valence (negative to positive, –3 to +3) and arousal (low to high, 0–6).

#### Fear of Pain

The Fear of Pain Questionnaire (FOPQ-C; (Simons et al., 2011)) is a self-report inventory to assess PRF. Each item is rated on a 5-point Likert-type scale from *0* = ‘*strongly disagree’* to *4* = ‘*strongly agree*.’ The FOPQ-C consists of 24 items with strong internal consistency (α = 0.92). The FOPQ-C has two subscales: Fear of Pain (α = 0.89) and Avoidance of Activities (α = 0.86). Construct validity for this measure is supported with significant relations found for the FOPQ-C with child somatization, anxiety, and catastrophizing. Criterion-related validity is also supported with significant relations between higher FOPQ-C scores and greater functional disability and more frequent doctor visits in the previous 3 months. Stability of the FOPQ-C total scale score is adequate (α = 0.74) with decreases in FOPQ-C scores associated with concomitant decreases in functional disability (*r* = 0.45) at 1-month follow-up, suggesting sensitivity to treatment response (Simons et al., 2011). High-fear patients (FOPQ ≥ 50) in this study were defined from the highest tertile in the validation sample (Simons et al., 2011).

### MRI Acquisition

Subjects underwent MRI on a 3 T (Siemens Medical Solutions, Erlangen, Germany) scanner using a 12-channel head coil. For each participant, we collected a 3D T1-weighted anatomical scan using a magnetization-prepared rapid acquisition gradient echo (MPRAGE) sequence (128 slices; TR = 2100 ms; TE = 2.74 ms; TI = 1100 ms; 256 × 256 matrix; FOV = 200 mm; 1.33 mm × 1.0 mm × 1.0 mm voxels). Two 5-min evoked fMRI scans were acquired using a T2^∗^-weighted echo-planar pulse imaging (EPI) sequence (41 interleaved slices; TR = 2500 ms; TE = 30 ms; 64 × 64 matrix; FOV = 1680 mm; 3 mm × 3 mm × 3 mm voxels; 120 volumes). Subjects were instructed to look carefully at the pictures and were told that they would be asked questions about them after the MRI.

### MRI Preprocessing and Data Analysis

#### Evoked Response to Fearful Faces

All preprocessing, first-level, second-level, and third-level group analyses were performed using FMRIB Software Library (FSL).

##### MPRAGE preprocessing

For each subject, MPRAGE images were skull-stripped using the brain extraction tool (BET).

##### fMRI preprocessing and first-level analysis

The following steps were taken within FEAT for each run of the evoked data set: (i) EPI images were skull-stripped using BET (Smith, 2002); (ii) motion correction using FMRIB’s Linear Motion Correction (MCFLIRT); (iii) spatial smoothing at 5 mm full-width at half maximum (FWHM); (iv) affine registration of the fMRI dataset to the Montreal Neurological Institute (MNI)-152 2 mm template brain using FMRIB’s Linear Image Registration Tool (Jenkinson and Smith, 2001; Jenkinson et al., 2002); (v) highpass filtering; and (vi) six standard motion parameters (i.e., three rotational and three translational) and a motion artifact confound matrix (created using FSL Motion Outliers for motion <3 mm) was added as variables of no interest. The fearful faces explanatory variable (EV) was constructed based on the temporal presentation of the images. The EV was convolved with standard gamma functions to produce a hemodynamic response model. We examined signal change relative to inter-interval fixation.

##### Second-level analysis

Once individual GLM FEAT analyses were completed, a fixed effects analysis was conducted combining both runs of the fearful faces paradigm to generate a mean image across runs.

##### Third-level analysis

The mean cope image for each individual was entered into an unpaired mixed-effects group analysis between patients and controls. For statistical thresholding, all whole-brain images were thresholded at *z* = 1.96 and cluster-wise corrected at *p* < 0.05 for multiple comparisons. These steps were replicated for the each facial expression condition (i.e., happy, fear, neutral).

#### ROI Analysis

To disentangle evoked brain response within patients by PRF level, we conducted region-of-interest (ROI) analysis using FSL Featquery for the ACC, dlPFC, amygdala (basolateral, centromedial regions), hippocampus, thalamus, putamen, caudate, and insula (anterior, posterior regions). Selection of these regions was based on results from recent work identifying these regions as having stronger synchronicity in activity with the amygdala at rest in pediatric CRPS (Simons et al., 2014b), as well as their known roles in fear learning (Linnman et al., 2012; Feng et al., 2014) and the emotional dimensions of pain (Neugebauer et al., 2009). Masks for the ROI analyses were created in FSL using Harvard-Oxford Cortical and Subcortical Atlas and Juelich Histological Atlas, and transformed into standard space, and thresholded at 50%. The insula from the Harvard-Oxford Cortical Atlas was subdivided into anterior and posterior regions at y = 1 (Brooks et al., 2005). Talairach Daemon Labels were also used to identify and create a dlPFC mask based on all voxels including Brodmann 9 and 46 regions within the middle frontal gyrus (MFG; Potkin et al., 2009). Percent signal change values represent mean values for the entire extracted ROI. Using one-way ANOVAs, we examined group differences between high-fear individuals (*n* = 8), non-elevated fear individuals (*n* = 8), and healthy controls (*n* = 19). Pearson Product Moment correlations were used to examine association between percent signal change and PRF level. All ROI analyses were *a priori* thresholded at *p* < 0.05.

#### Age-Related Activation

Given that age-related changes in activation have been observed for amygdala-PFC connectivity (Gee et al., 2013) and that the patient and healthy control sample were age-matched, we examined whether the linear relationship between evoked activation and age differed between the two groups using a continuous covariate interaction where mean centered age is entered into two EVs (one for patients, one for controls).

## Results

### Participants

A total of 20 patients and 20 age- and sex-matched healthy controls completed the emotional faces paradigm. All study participants were right-handed. Four study participants (three patients, one control) displayed excessive motion (>3 mm) and one did not have a visual cortex response across emotional stimuli (one patient) resulting in a final group of 16 patients and 19 controls. All subsequent descriptive numbers reflect this group of patients and healthy controls. Among patients, 75% were female with an average age ± SD of 13.7 ± 3.2 years, and as expected, not significantly with the matched healthy controls (79% female; mean age = 14.1 ± 3.1 years). Within patients, 44% were right-side aﬄicted, 44% left-side aﬄicted, and 12% presented with bilateral lower extremity CRPS diagnosis. With regards to current medications, 38% (*n* = 6) were prescribed antiepileptic medication (e.g., gabapentin), 12% (*n* = 2) was prescribed an antiepileptic medication and a tricyclic, 6% (*n* = 1) was prescribed a muscle relaxant, while all other patients (*n* = 8) were not currently prescribed any pain medications. Three patients met criteria for an Axis I psychiatric diagnosis (*n* = 2 had generalized anxiety and *n* = 1 had generalized anxiety and major depression).

### Pain and PRF

Among the 16 patients, 81.3% of patients self-reported moderate to severe average pain levels (average pain intensity ± SE = 6.17 ± 0.61). Median duration of pain was 7 months (average pain duration = 13.25 ± 3.87). For PRF, 50% (*n* = 8) reported high PRF (average PRF = 48.06 ± 3.77). PRF and pain intensity were not statistically significantly associated with one another (*r* = –0.20; *p* = 0.45).

### Facial Expression Ratings

**FIGURE 2:**
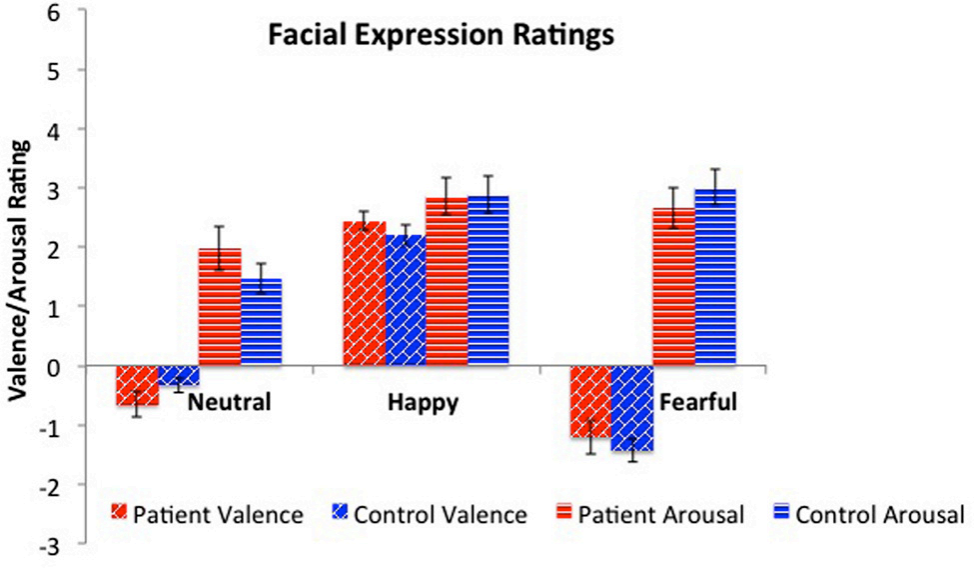
**Valence and arousal ratings across facial expressions.** Patients and healthy peers did not differ on valence or arousal ratings for neutral, happy, and fearful faces. As expected, there were significant differences across emotions for valence, with fearful faces rated more negatively and happy faces rated more positively. For arousal, both fearful and happy faces were rated as significantly more arousing than neutral faces.

Ratings of valence and arousal were submitted to separate 2×3 (group [patient vs. healthy control] × expression [neutral, happy, fear]) analyses of variance. For valence, there was a significant main effect for expression (*F*_2,102_ = 189.7, *p* < 0.01), but not for group (see **Figure 2**). *Post hoc* testing showed significant valence rating differences among all three types of facial expressions; fearful (-1.34 ± 0.16) vs. happy (2.31 ± 0.12), *t*(33) = –16.7, *p* < 0.01; fearful vs. neutral (–0.47 ± 0.12), *t*(33) = –6.00, *p* < 0.01, and neutral vs. happy, *t*(33) = –15.3, *p* < 0.01. Analysis of the arousal ratings revealed a significant main effect for expression (*F*_2,102_ = 8.69, *p* < 0.01), but not for group. *Post hoc* testing showed significantly lower arousal ratings for neutral faces (1.70 ± 1.27) vs. fearful (2.90 ± 1.33), *t*(33) = –5.76, *p* < 0.01, and happy (2.87 ± 1.29), *t*(33) = –5.72, *p* < 0.01. Arousal ratings between happy and fearful did not significantly differ.

### fMRI Results

#### Fearful Faces

**FIGURE 3:**
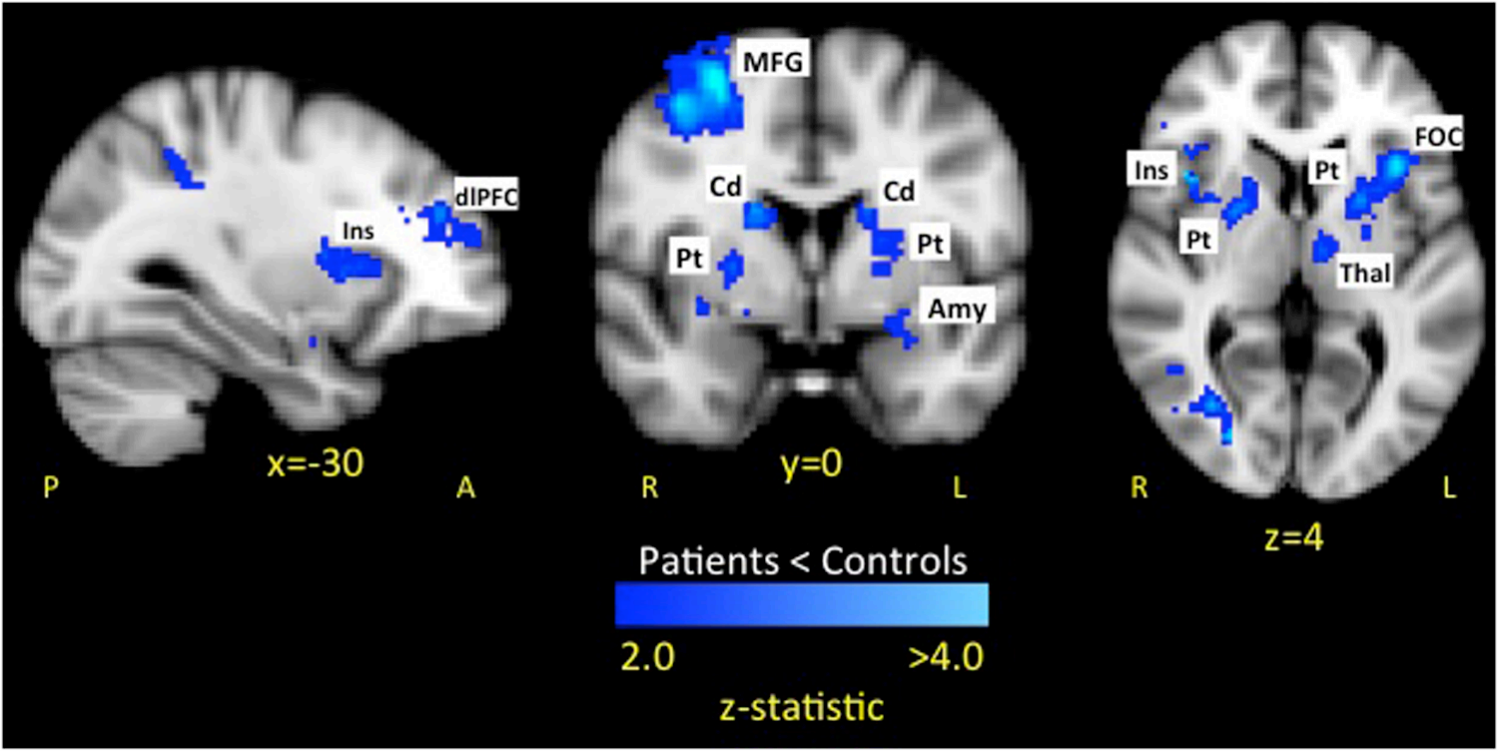
**Dampened response to fearful expressions in pediatric CRPS patients compared to healthy peers.** The attenuated response among patients to fearful expressions was observed in key limbic and prefrontal circuits. No areas were observed to have greater activation in patients compared to controls. Key: Amygdala (Amy), caudate (Cd), dorsolateral prefrontal cortex (dlPFC), frontal operculum cortex (FOC), insula (Ins), putamen (Pt), middle frontal gyrus (MFG).

The whole brain analysis revealed six significant clusters with less evoked activation for patients compared to healthy controls in response to fearful faces (see **Figure 3** and **Table 1**). The first cluster was in the right hemisphere consisting of 1518 voxels including the frontal operculum cortex (FOC), insula cortex (Ins), and striatum (Caudate [Cd], Pallidum [Pd], Putamen [Pt]). The second cluster was in the left hemisphere consisting of 1375 voxels including the FOC, Ins, dlPFC, MFG, thalamus (thal), and striatum (Cd, Pt). The third cluster was in the left hemisphere consisting of 1236 voxels including the lateral occipital cortex (LOC), superior parietal lobule (SPL), supramarginal gyrus (SMG), and postcentral gyrus (PoCG). The fourth cluster was in the right hemisphere consisting of 976 voxels including the superior frontal gyrus (SFG), MFG, and precentral gyrus (PrCG). The fifth cluster was in the right hemisphere consisting of 975 voxels including the SPL and LOC. The sixth cluster was in the right hemisphere consisting of 858 voxels including the PrCG, inferior frontal gyrus (IFG), SMG, PoCG. No areas were observed to have greater activation in patients compared to controls.

**Table 1:** Evoked response to overt fearful faces: Patients < Healthy Controls.

Brain Region		MNI Coordinates			*z-stat*
	Voxels	*x*	*y*	*z*	
**Cluster 1**	1518				
Cortical: Right Insula					
Frontal Operculum Cortex		42	16	4	3.38
Insular Cortex		30	16	10	3.24
Subcortical: Right Striatum					
Caudate		18	–2	22	3.29
Pallidum		28	–16	–2	3.14
Putamen		24	2	2	3.04
		32	–16	–2	3.02
**Cluster 2**	1375				
Cortical: Left Frontal, Left Insula					
Frontal Operculum Cortex/Insula		–36	18	6	3.63
Dorsolateral Prefrontal Cortex (dlPFC)		–42	48	28	3.23
Middle Frontal Gyrus		–40	36	26	3.01
Subcortical: Left Thalamus, Left Striatum					
Thalamus		–14	–8	12	3.28
Putamen		–20	4	8	3.25
Caudate		–16	–2	20	3.15
**Cluster 3**	1236				
Cortical: Left Parietal/Occipital					
Lateral Occipital Cortex		–20	–58	48	3.46
		–20	–64	52	3.42
Superior Parietal Lobule		–24	–46	38	3.31
Supramarginal Gyrus		–58	–32	50	3.06
		–50	–30	44	2.93
Postcentral Gyrus/S1		–44	–34	48	3.00
**Cluster 4**	976				
Cortical: Right Frontal/Motor Cortex					
Superior Frontal Gyrus		28	2	60	4.01
		24	4	52	3.31
Middle Frontal Gyrus		30	8	56	3.78
		30	–4	54	3.48
		40	0	52	3.25
Precentral Gyrus		28	–6	44	3.27
**Cluster 5**	975				
Cortical: Right Parietal/Occipital					
Superior Parietal Lobule		28	–56	50	4.19
		30	–56	58	3.80
Lateral Occipital Cortex		30	–58	62	3.46
		32	–64	62	3.40
		26	–60	58	3.27
		30	–60	58	3.26
**Cluster 6**	858				
Right Frontal/Parietal/Motor Cortex					
Precentral Gyrus		54	10	30	3.33
		50	6	32	3.12
		34	10	28	2.95
Inferior Frontal Gyrus		56	14	30	3.10
Supramarginal Gyrus		66	–32	28	2.88
Postcentral Gyrus		66	–16	24	2.79

*MNI coordinates and *z*-values are from the peak. Images were cluster-wise corrected at *p* < 0.05.*

#### Happy Faces

**FIGURE 4:**
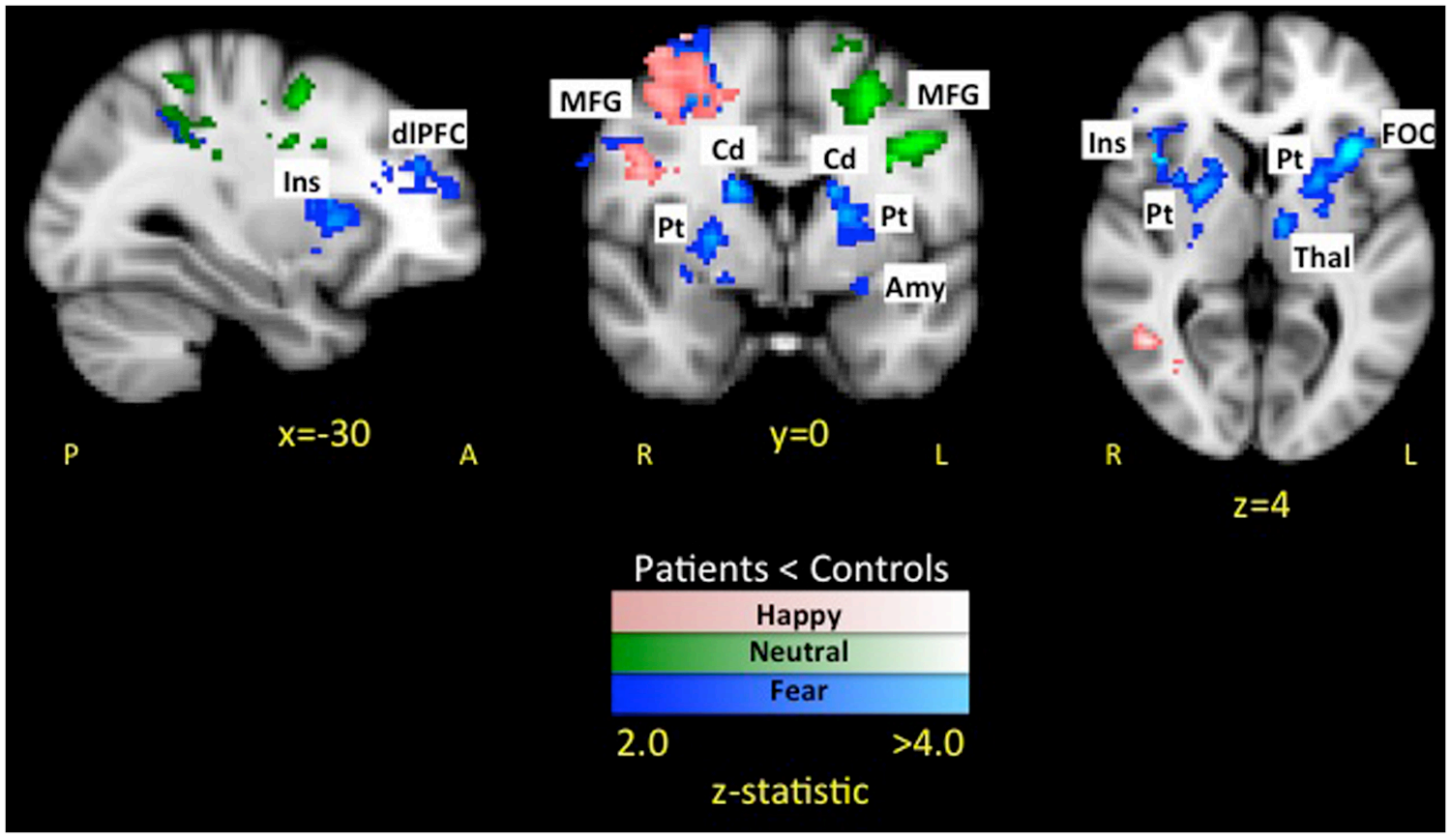
**Dampened response to fearful, happy, and neutral expressions in pediatric CRPS patients compared to healthy peers.** As depicted in the figure, there was overlap in differences between patients and healthy controls in response to happy faces in the left middle frontal and precentral gyrus, while overlap between fear and neutral faces was in the parietal lobe. Notably, differences in activation in striatal and insular regions were unique to the fearful facial expressions.

The whole brain analysis resulted in two significant clusters with less evoked activation for patients compared to healthy controls in response to happy faces (see **Table 2** and **Figure 4** activation noted in pink). The first cluster was in the right hemisphere consisting of 1265 voxels including the SFG, PrCG, and MFG. The second cluster was also in the right hemisphere consisting of 1002 voxels spanning over the angular gyrus, inferior temporal gyrus, SMG, and middle temporal gyrus. No areas were observed to have greater activation in patients compared to controls.

**Table 2:** Evoked response to overt happy faces: Patients < Healthy Controls.

Brain Region		MNI Coordinates			*z-stat*
	Voxels	*x*	*y*	*z*	
**Cluster 1**	1265				
Cortical: Right Frontal/Motor Cortex					
Superior Frontal Gyrus		28	–4	54	2.93
Precentral Gyrus		54	10	30	3.36
		28	–6	44	3.06
Middle Frontal Gyrus		46	20	32	2.84
		44	2	62	2.83
**Cluster 2**	1002				
Cortical: Right Temporal/Parietal					
Angular Gyrus		60	–58	14	3.63
Inferior Temporal Gyrus		50	–52	–14	3.47
		46	–56	–8	3.12
Supramarginal Gyrus		44	–38	20	3.20
Middle Temporal Gyrus		48	–50	8	3.16
		48	–44	8	3.14

*MNI coordinates and *z*-values are from the peak. Images were cluster-wise corrected at *p* < 0.05.*

#### Neutral Faces

In the whole brain analysis, two significant clusters emerged with less evoked activation for patients compared to healthy controls in response to neutral faces (see **Table 3** and **Figure 4** activation noted in green). The first cluster was in the left hemisphere consisting of 1064 voxels including the PrCG, SFG, and MFG. The second cluster was also in the left hemisphere consisting of 907 voxels including the PoCG and SPL. No areas were observed to have greater activation in patients compared to controls.

**Table 3:** Evoked response to overt neutral faces: Patients < Healthy Controls.

Brain Region		MNI Coordinates			*z-stat*
	Voxels	*x*	*y*	*z*	
**Cluster 1**	1064				
Cortical: Left Frontal/Motor Cortex					
Precentral Gyrus		–34	0	30	3.20
		–44	0	36	3.18
		–34	–4	34	3.01
Superior Frontal Gyrus		–24	4	50	3.14
Middle Frontal Gyrus		–26	–3	48	3.14
**Cluster 2**	907				
Cortical: Left Parietal Lobe					
Postcentral Gyrus		–22	–44	54	3.25
Superior Parietal Lobule/S1		–26	–40	52	3.19
		–26	–48	52	3.12
		–36	–42	42	3.02
		48	–50	8	3.16

*MNI coordinates and *z*-values are from the peak. Images were cluster-wise corrected at *p* < 0.05.*

#### Female Only Results

When limiting the analyses to only female patients (*n* = 12), the majority of results were replicated. For fearful faces, differences in the left dlPFC, left MFG, and left parietal and occipital cortex were no longer significant. All other differences were replicated. For happy faces, the cluster in the right frontal/motor region was no longer significant, while the cluster in the temporal region remained significant. For neutral faces, no clusters emerged as significant. Consistent with the whole sample analysis, there were no areas where greater activation was observed in female patients compared to female controls across all emotional contexts.

#### Habituation Results

We combined both runs of the fearful, happy, and neutral face paradigms to generate a mean image across runs to increase our statistical power and reliability of results. As affective responding could have potentially differentially attenuated between patients and controls, we examined these differences. We found that patients had less habituation in brainstem regions to fearful faces compared to healthy peers (cluster max: *z* = 3.33, *x* = –8, *y* = –58, *z* = –46. No differences in habituation emerged for happy or neutral faces.

#### High-Fear vs. Non-Elevated Fear vs. Healthy Control

**FIGURE 5:**
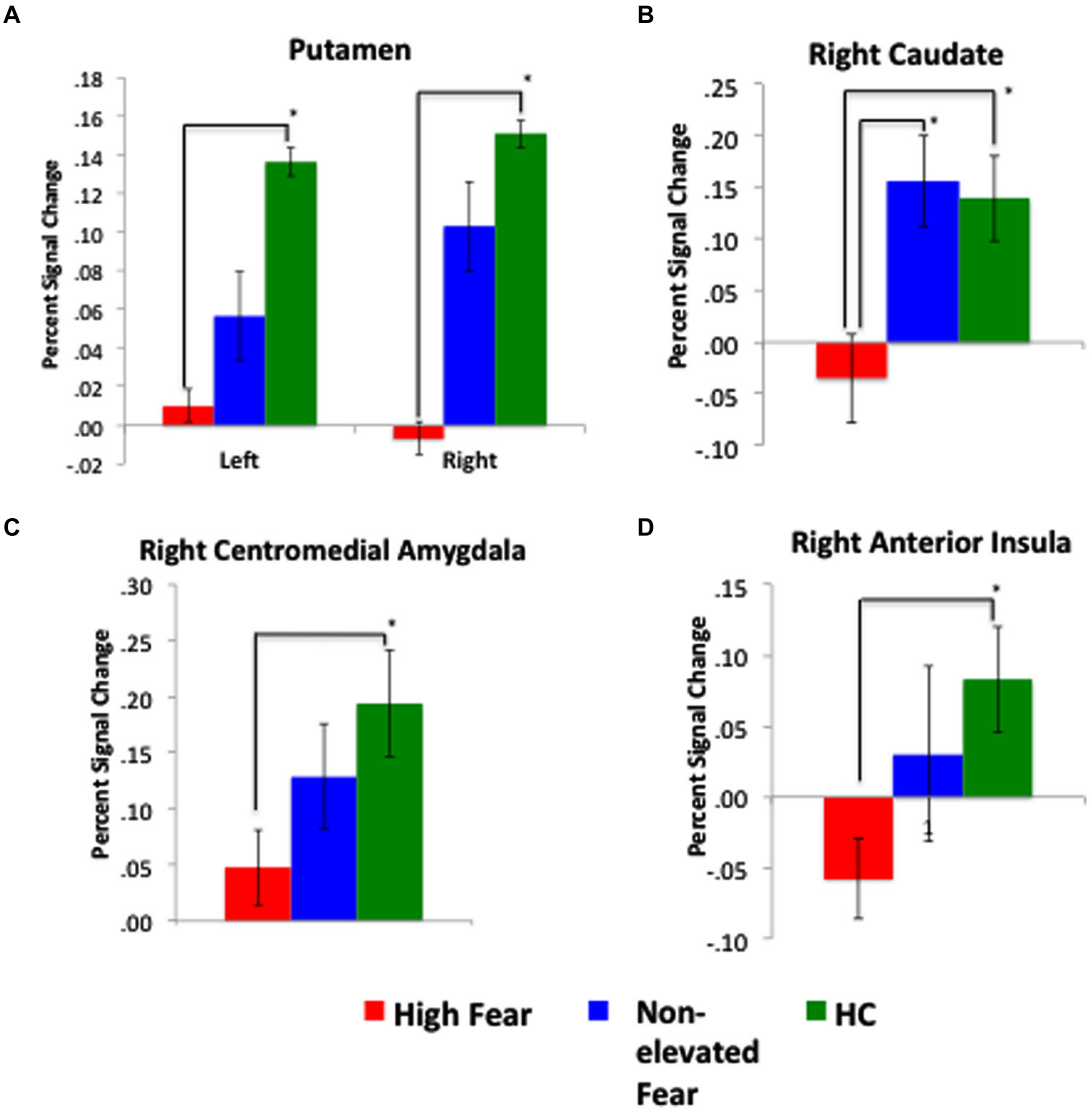
**Response to fearful faces most attenuated among high-fear patients.** Across the four figures: **(A)** depicts a significant difference in percent signal change in the left and right putamen between high-fear patients and healthy controls (HC); **(B)** depicts a significant difference in the right caudate across all three groups; **(C)** displays a significant difference in the right centromedial amygdala between high-fear patients and HCs; and **(D)** displays a significant difference in the right anterior insula between high-fear patients and HCs. Percent signal change values represent mean values for the entire extracted ROI.

**FIGURE 6:**
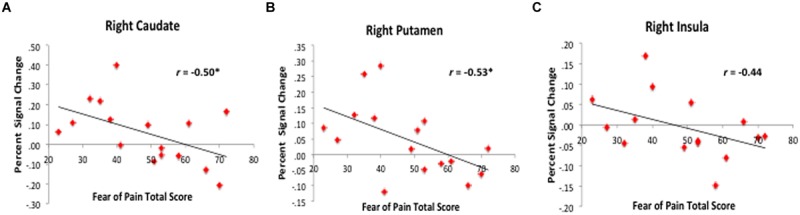
**Correlation between fear of pain level and evoked response to fearful faces.** Among patients, higher fear of pain scores were associated with less activation or deactivation in the right caudate **(A)**, putamen **(B)**, and insula **(C)**. Percent signal change values represent mean values for the entire extracted ROI.

To disentangle evoked brain response within patients by PRF level, we conducted ROI analysis using FSL Featquery. We observed that high-fear patients (*n* = 8) had less activation in the bilateral Pt and right Cd, right centromedial amygdala, and right anterior Ins (see **Figure 5**) compared to non-elevated fear (*n* = 8) and healthy controls (*n* = 19). When examining the correlation between fear of pain level and percent signal change (*n* = 16), higher PRF scores were associated with attenuated responses in the right Cd (*r* = –0.50, *p* = 0.05), right Pt (*r* = –0.53, *p* = 0.04), and right Ins (*r* = –0.44, *p* = 0.10; see **Figure 6**). Although high fear patients had less activation in the left putamen and right centromedial amygdala compared to healthy controls, the correlation between PRF and percent signal change within patients was not significant; suggesting that the differences in these brain areas was likely most driven by high fear status. The magnitude of the correlations did not change when pain level and pain duration were included as covariates. These results suggest that beyond differences between healthy individuals and patients with chronic pain, the level of PRF is associated with altered cognitive-affective brain circuit responses to emotionally relevant stimuli.

#### Age-Related Activation

**FIGURE 7:**
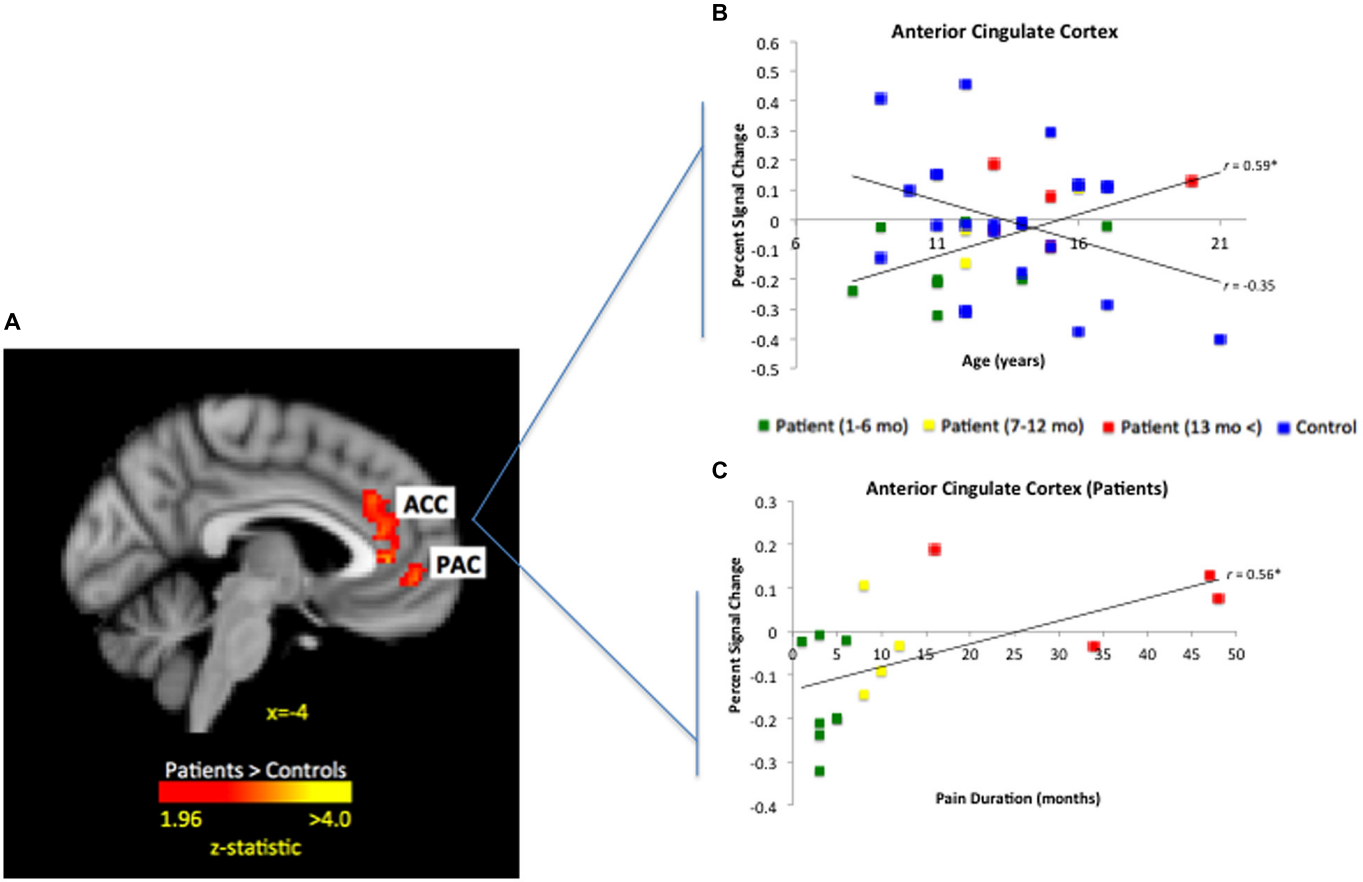
**Significant interaction for age by group for fearful faces in the ACC.** As depicted in **(A)** patient slope was significantly greater than healthy control slope for age in the ACC and PAC (peak activation MNI*_x,y,z_* = 4, 24, 20; *z*-score = 3.51; *p* < 0.05, corrected; 948 mm^3^). When plotting out individual values as seen in **(B)**, controls showed a general decrease in ACC response across age, whereas patients had a significant increase in ACC response. As displayed in **(C)**, ACC activation was significantly associated with pain duration among patients. Those who had experienced pain for 6 months or less had ACC deactivation in response to fearful faces whereas patients with greater than 1 year of pain having greater ACC activation.

A continuous covariate interaction across patients and healthy controls was examined in relation to age, given the potential influence of age and development on evoked brain responses to fearful faces. The interaction was significant for the ACC and paracingulate gyrus (PAC) with patient slope greater than healthy controls (see **Figure 7A**); in other words, for patients, evoked ACC responses significantly increased across age while responses in healthy control showed a non-significant decrease across age (see **Figure 7B**). This decreasing response in healthy participants is consistent with prior work looking at developmental changes in response to fearful stimuli in the ACC (Monk et al., 2003). Given the potential confound of pain duration on patients’ age, we found that pain duration was significantly associated with ACC activation (see **Figure 7C**). In a partial correlation analysis controlling for pain duration, increased activation was no longer statistically associated with older age, with the magnitude of the association medium in size (*r* = 0.38; *p* = 0.08), thus persistent disease state appears to be partially driving increased activation within the ACC across age.

## Discussion

Here we report the first data among pediatric CRPS patients examining brain responses to an experimental paradigm designed to engage emotional circuits and produce an affective state. We observed decreased evoked responses to fearful stimuli in patients with CRPS compared to healthy sex- and age-matched controls in prefrontal and limbic regions, with salient differences in the striatum, amygdala, Ins, and dlPFC. In addition, we found that the blunted response to fearful expressions in the Cd, Pt, centromedial amygdala, and anterior Ins was associated with PRF levels. These results corroborate accumulating research that has demonstrated alterations in cognitive-affective brain regions in the chronic pain state (Lebel et al., 2008) and may reflect allostatic over-load, thus dampening an important adaptive response to threatening (fearful) stimuli (McEwen and Kalia, 2010; Karatsoreos and McEwen, 2011). Self-reported emotional arousal and valence to happy, fearful, and neutral faces were in the expected direction with both happy and fearful faces more emotionally arousing than the neutral faces and the fearful faces rated more negatively than the neutral and happy faces. These ratings did not differ between patients and healthy controls, suggesting that the differences observed here are implicit, rather than explicitly reported. Overall our findings provide novel evidence that pediatric patients with chronic pain show altered processing of negative affective information, which may either serve as a vulnerability factor or contribute to pain persistence.

### CNS Processing of Fear in CRPS

Previous studies have demonstrated an increased response in limbic areas during fear processing in patients with PTSD (Shin et al., 2005), social anxiety (Demenescu et al., 2013), and specific phobia (Etkin and Wager, 2007), with decreased response observed in panic (Pielech et al., 2014), thus we anticipated an altered evoked response in the amygdala among chronic pain patients compared to healthy peers, with this result amplified among high-fear patients compared to patients with non-elevated fear. We found that high PRF patients had a reduced response in the amygdala, potentially suggesting that as a result of ongoing aversive sensory processes, fear-related emotional circuits in chronic pain patients may be altered. Several processes may account for these results. First, other factors that contribute to pain such as the aversive nature of pain itself, as well as salience and reward processes, and stress may elicit alterations in multiple brain circuits. Additionally, patients may be so distressed by their own pain state that they are limited in their ability to empathize or connect with another’s emotional state due to the self-focused state driven by persistent pain signaling (Ochsner et al., 2006), therefore potentially resulting in a blunted response in fear-related emotional circuits.

Among the specific findings, we observed significant differences between patients and controls in the centromedial amygdala. This area has been implicated in generating behavioral responses to fearful stimuli through projections to the brainstem, as well as cortical and striatal regions, like the Cd (LeDoux, 2007). The amygdala has an important role and is significantly altered morphologically and structurally in chronic pain (Rodriguez-Raecke et al., 2009). Amygdala volume has been observed to be decreased in chronic pain (Rodriguez-Raecke et al., 2009), with our prior work in CRPS patients showing increased amygdala volume following treatment with reversal of pain symptoms (Erpelding et al., 2014), suggesting a role in both aversive behaviors (chronic pain) and rewarding effects (pain relief; Janak and Tye, 2015). As a key brain region for fear circuitry (Herry and Johansen, 2014), the amygdala may contribute to functional changes through connections that are altered in chronic pain including pediatric patients (Becerra et al., 2014; Erpelding et al., 2014; Simons et al., 2014b) and in the context of ongoing stress (Ressler, 2010). Dampened activity in the amygdala in patients compared to healthy matched controls may arise from emotional processing deficits, as having an evoked response to fearful stimuli in this region is adaptive. Additionally, persistent pain may hinder proper functioning activity in the centromedial amygdala and may modulate pain behavior through signals sent to descending pain control centers in the brain (Neugebauer et al., 2009).

Furthermore, prominent differences between patients and healthy controls were observed in the striatum and anterior Ins. Striatal regions are significantly involved in pain processing (Borsook et al., 2010; Maleki et al., 2011), and specifically, thought to integrate sensory, emotional, and cognitive processing. The Cd is also known to participate in learning and memory (Chase et al., 2015) and goal-directed behavior (Yanike and Ferrera, 2014). For example, fibromyalgia patients exhibit reduced activation in the Cd and hippocampus in response to the Stroop task (Martinsen et al., 2014). A recent study examined learning PRF and found that successful aversive learning was associated with activation in the Pt, Ins, and secondary somatosensory cortex (S2) (Gramsch et al., 2014). Additionally, less activation to pain in the Cd and Pt has been observed in high-frequency migraine compared to low-frequency migraine patients (Maleki et al., 2011). Our finding of differences in the bilateral anterior Ins, a region involved in the salience network (Uddin, 2015) and autonomic function, is tied to multiple fear-related adaptations. It is conceivable that altered salience in chronic pain reflects diminished awareness and responses to external stimuli unrelated to pain, which may be due to a perceived alteration in the body’s integrity (e.g., hemi-inattention; Forderreuther et al., 2004). A number of observations suggest that chronic pain patients exhibit abnormal salience processing (Seeley et al., 2007; Cauda et al., 2010; De Ridder et al., 2011; Weissman-Fogel et al., 2011). Beyond differences in key subcortical regions and the Ins, less response in the dlPFC was observed among patients compared to healthy controls. The dlPFC has a prominent role in top-down cognitive control in the context of emotion (Buhle et al., 2014) and pain (Erpelding and Davis, 2013) modulation. In addition to the well-established link between the amygdala and the dlPFC, it is structurally and functionally connected to the striatum (Jarbo and Verstynen, 2015). Taken together, the blunted response observed in patients appears to be present across learning and reward circuits.

When examining these findings in relation to imaging work conducted among adults with CRPS, the majority of brain imaging research among adults with CRPS has focused on somatosensory and motor cortex alterations (e.g., Di Pietro et al., 2013), although one recent study did find altered gray matter structure in the dlPFC (Pleger et al., 2014). Research comparing pediatric and adult patients with CRPS is needed.

### Influence of Age in Response to Fearful Faces

Age and its interaction with brain activity has not been previously examined in pediatric pain, but given that fear-related neural activity has been observed in the PFC (Yurgelun-Todd and Killgore, 2006), we conducted a covariate interaction analysis by age. We found that the functional response in the ACC was greater with increased patient age, and was partially explained by pain duration. The ACC has an instrumental role in pain unpleasantness and PRF memory (Tang et al., 2005). It appears that a persistent pain state does relate to an increased evoked ACC response to fearful affective stimuli in contrast to healthy controls who demonstrate a general decrease in response across age. Importantly, no other brain regions emerged as differing between patients and controls by age, suggesting that the primary findings are not influenced by the child’s age. Accordingly, our findings suggest that altered fear circuits observed among pediatric CRPS can be attributed to disease state versus development.

### Caveats

Our study has a number of caveats: (1) *Loss of patients due to eligibility, decline, and motion* – Imaging in pediatrics has significant challenges. The majority of patients recruited for this study were being evaluated for our intensive pain rehabilitation program, thus multiple patients had medical or mental health comorbidities or were feeling overwhelmed with the upcoming treatment program, thus declined involvement in the study. Additionally, we faced challenges associated with scanning youth (metal dental work, motion). Despite these challenges, understanding brain alterations in youth with chronic pain is important and conclusions based on research among adults cannot serve as a replacement. (2) *Sex* – Although our patient sex distribution is reflective of the literature (de Mos et al., 2007), the preponderance of females were included in the study. Although females suffer more frequently from CRPS (Low et al., 2007), our sample limits generalizability of our results to young males suffering from CRPS and did not allow us to examine potential sex differences. (3) *Medication* – Half of the patients in the study were not currently taking any prescribed medication, while half of the patients were taking a pain medication (the majority being an anti-epileptic taken at bedtime), this split pattern was consistent for the patients classified as having high PRF. We do not have strong evidence of the impact of these medications on brain activity, but importantly none of the patients were taking narcotics, which we do know has a significant impact on the brain.

## Conclusion

Chronic pain is multifaceted with clear physiological, cognitive, and affective dimensions. This study provides novel evidence that pediatric patients with CRPS demonstrate altered corticolimbic circuit response in fear perception that appears to be driven or maintained by PRF levels for the striatum, centromedial amygdala, and anterior Ins. Our results suggest that the dampened response could reflect altered learning, memory, and attention in the context of a persistent and debilitating pain state. Clinically elevated PRF may reflect maladaptive aversive learning that is resistant to extinction. The dampened response observed in brain regions such as the Ins and Pt in this study may constitute important neural markers for deficit learning that leads to a pathological PRF level. Accordingly, these brain function alterations may either serve as a vulnerability factor or contribute to the persistent pain state and warrant further study.

## Author Contributions

All authors contributed significantly to this work. All authors have read and reviewed this manuscript and agree on its submission to Frontiers in Behavioral Neuroscience.

## Conflict of Interest Statement

The authors declare that the research was conducted in the absence of any commercial or financial relationships that could be construed as a potential conflict of interest.
